# Molecular Analysis of Precursor Lesions in Familial Pancreatic Cancer

**DOI:** 10.1371/journal.pone.0054830

**Published:** 2013-01-23

**Authors:** Tatjana Crnogorac-Jurcevic, Claude Chelala, Sayka Barry, Tomohiko Harada, Vipul Bhakta, Sam Lattimore, Stipo Jurcevic, Mary Bronner, Nicholas R. Lemoine, Teresa A. Brentnall

**Affiliations:** 1 Molecular Oncology Centre, Barts Cancer Institute, Queen Mary University of London, London, United Kingdom; 2 Division of Transplantation Immunology & Mucosal Biology, King's College, London, United Kingdom; 3 Department of Anatomic Pathology, University of Utah, Salt Lake City, Utah, United States of America; 4 Department of Medicine, Division of Gastroenterology, University of Washington, Seattle, Washington, United States of America; Technische Universität München, Germany

## Abstract

**Background:**

With less than a 5% survival rate pancreatic adenocarcinoma (PDAC) is almost uniformly lethal. In order to make a significant impact on survival of patients with this malignancy, it is necessary to diagnose the disease early, when curative surgery is still possible. Detailed knowledge of the natural history of the disease and molecular events leading to its progression is therefore critical.

**Methods and Findings:**

We have analysed the precursor lesions, PanINs, from prophylactic pancreatectomy specimens of patients from four different kindreds with high risk of familial pancreatic cancer who were treated for histologically proven PanIN-2/3. Thus, the material was procured *before* pancreatic cancer has developed, rather than from PanINs in a tissue field that already contains cancer. Genome-wide transcriptional profiling using such unique specimens was performed. Bulk frozen sections displaying the most extensive but not microdissected PanIN-2/3 lesions were used in order to obtain the holistic view of both the precursor lesions and their microenvironment. A panel of 76 commonly dysregulated genes that underlie neoplastic progression from normal pancreas to PanINs and PDAC were identified. In addition to shared genes some differences between the PanINs of individual families as well as between the PanINs and PDACs were also seen. This was particularly pronounced in the stromal and immune responses.

**Conclusions:**

Our comprehensive analysis of precursor lesions without the invasive component provides the definitive molecular proof that PanIN lesions beget cancer from a molecular standpoint. We demonstrate the need for accumulation of transcriptomic changes during the progression of PanIN to PDAC, both in the epithelium and in the surrounding stroma. An identified 76-gene signature of PDAC progression presents a rich candidate pool for the development of early diagnostic and/or surveillance markers as well as potential novel preventive/therapeutic targets for both familial and sporadic pancreatic adenocarcinoma.

## Introduction

Pancreatic cancer is the fourth leading cause of cancer death in the United States and its frequency has been rising in recent years [1]. Due to lack of clinically overt symptoms the majority of patients present with a disseminated disease and are largely incurable. The abysmally low survival rate could be greatly improved by effective methods of early detection, while cancer is still surgically curable, with a ‘window of opportunity’ for the timely diagnosis (e.g. the pre-metastatic stage of cancer) being, according to a recent report, more than a decade [2]. Such diagnostic methods will almost certainly include molecular analysis, and yet very few large-scale studies to investigate the process of early development of pancreatic cancer have been undertaken.

A widely accepted paradigm is that PDAC develops through series of precursor lesions called PanINs (pancreatic intraepithelial neoplasia). Based on the degree of cellular and nuclear atypia, these lesions progress from PanIN-1, characterized by hyperplastic columnar ductal epithelia with no nuclear atypia, through PanIN-2, that displays low-grade dysplasia, to PanIN-3 (carcinoma *in situ*), which shows high-grade dysplasia [3]. The linearity of this progression is still unclear, although, based on several reports which show frequent PanIN-1 lesions in otherwise healthy people and taking into account the low prevalence of PDAC associated with PanIN-1, these early stage lesions are probably indolent in nature. In contrast, based on available molecular data, PanIN-2 and -3 lesions are highly likely to be the true PDAC precursors [4], [5].

A major obstacle for the detailed study of PDAC evolution is obtaining clinical material from PanIN lesions, which is a particularly daunting task since the patients are largely asymptomatic and these ductal changes are typically focal. In fact, PanIN-2 and -3 lesions are usually random findings in pathological sections of specimens with frank malignancy and are often not recorded in routine histopathological evaluation.

In this study, in order to reconstruct the natural history of the disease, we analysed fresh frozen pancreatic tissue that had dysplastic PanIN-2 and focal PanIN-3 lesions as the most advanced histological abnormalities in the pancreas, without an accompanying cancer. This is critical as use of dysplastic lesions from adenocarcinoma cases could risk the inclusion of field defects and duct cancerisation that are absent in specimens which are still cancer-free. Such material would not be randomly available; it was obtained from high-risk patients who inherit pancreatic cancer and are participating in a cancer surveillance program developed at the University of Washington [6], [7].

The study of families in which cancer is inherited in an autosomal dominant fashion has provided considerable insight into the molecular basis of the disease; inherited pancreatic cancers represent up to 10% of all pancreatic cancers [8], [9]. We have analysed four familial pancreatic cancer (FPC) cohorts, Family X [10] and three additional pedigrees (here termed non-X families) and contrasted them with normal pancreas and sporadic pancreatic cancer. Family X has a rare, highly penetrant, autosomal dominant form of FPC that is characterised by a germline mutation in the palladin gene, an embryonic protein that regulates cell motility and invasion [11]. The non-X FPC families were a heterogeneous population with unknown germline mutational status; no mutations were detected in CDKN2A (data not shown) and further testing for BRCA2, PALB2 and ATM was not performed due to the general low prevalence of these gene mutations in FPC and the insufficient amount of material.

The transcriptomic, and proteomic [12] profiling of advanced human PanINs from such families is critical for revealing the molecular changes underlying the progression towards PDAC and might provide a framework for devising novel surveillance, preventive, and treatment modalities.

## Results

**Figure 1:**
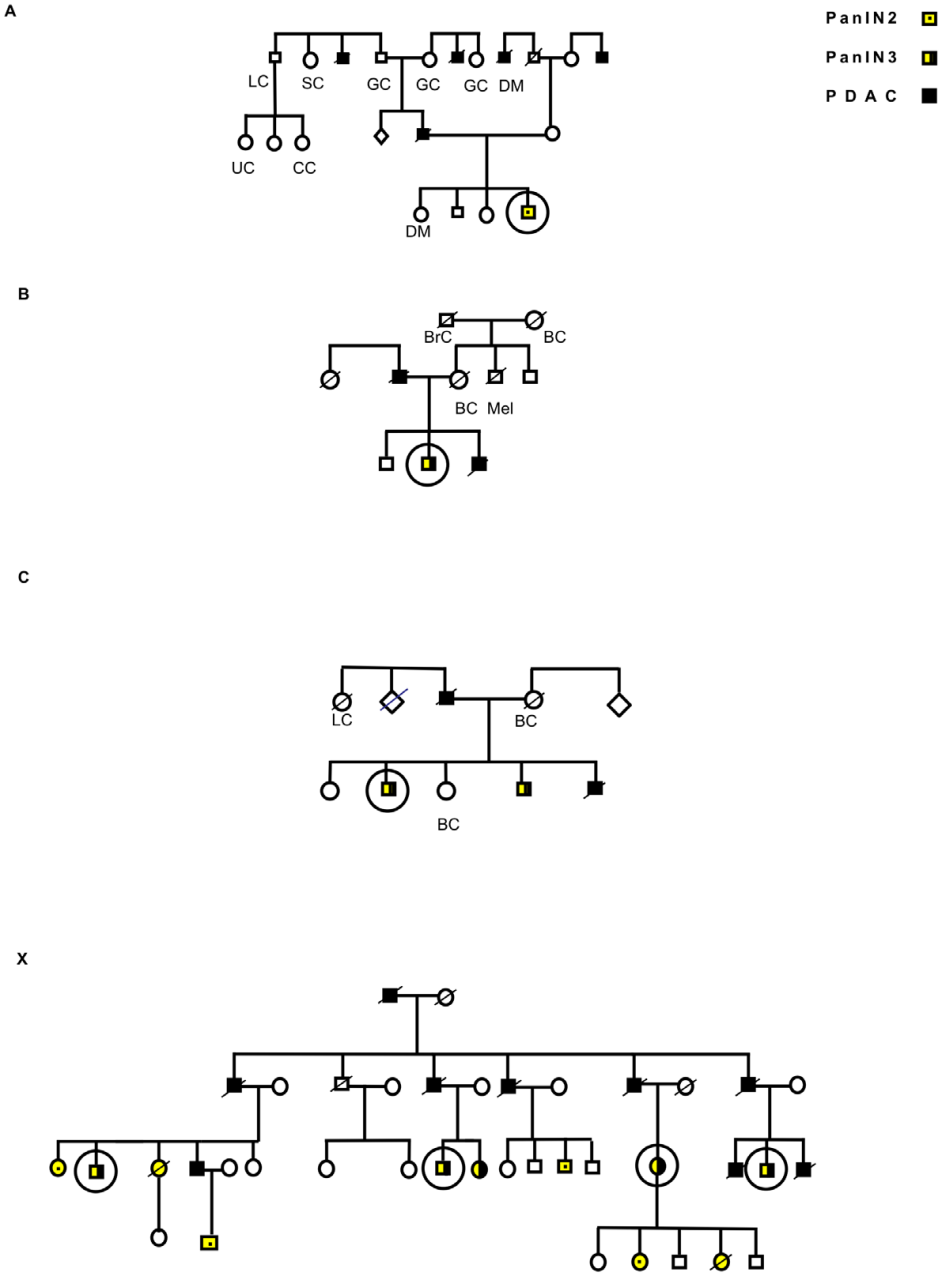
The pedigrees of pancreatic cancer families analysed. (**A–C**) non-X families and (X) Family X.

The pedigrees of four different FPC families with at least two affected members are shown in **Figure 1**. Three smaller kindreds (A, B and C) have in addition to PDAC also other solid malignancies, while Family X (X), which is characterised by early onset disease and often preceded by endocrine (Diabetes mellitus) and exocrine insufficiency [10], [13] is affected only by PDAC. The patients from which samples were obtained are circled in **Figure 1**; patients' clinical information is summarized in **Table 1**.

**Table 1:** Clinical information.

Sample	Pedigree	Gender	DOB	Age at surgery	Smoking	Alcohol	DM	Hg A1c	Symptoms	Years of EUS	Years of FU	EUS	ERCP	Highest histology finding	Surgery	Age range of PDAC onset in family	Number of family members with PDAC
**Non-X Families**																	
PanA1	III.4	M	1953	49	no	moderate	no	5.1	steatorrhea	5	5	Mild CP	Narrow duct	PanIN2	partial	64-NA	5M, 0F
PanB1-3	III.2	M	1940	62	remote	no	no	5.8	none	5	5	Abn	Abn	PanIN3	total	53–66	2M, 0F
PanC1-2	II.2	M	1953	49	remote	no	no	NA	none	2	6	Nl to Abn 1y later	Abn	PanIN3	total	50–58	4M, 0F
**Family X**																	
PanX1-3	III.8	M	1959	41	yes	heavy	AODM	NA	loose stools	1	11	Abn	Abn	PanIN3	total	28–57	9M, 0F
PanX4	III.16	M	1956	42	previously	moderate	AODM	13.6	loose stools	1	8	Abn	Abn	PanIN3	total	28–57	9M, 0F
PanX5,7	III.2	M	1956	41	yes	yes	IDDM	9.4	loose stools	2	11	Nl to Abn 1y later	Abn	PanIN3	total	28–57	9M, 0F
PanX6	III.14	F	1943	53	yes	heavy	AODM	7.7	loose stools, weight loss	1	11	Abn	Abn	PanIN3	total	28–57	9M, 0F

M = male; F = female; HgA1c = Hemoglobin A1c (normal range 4.0–6.0); IDDM = insulin dependent diabetes mellitus; AODM = adult onset diabetes mellitus; NA = not available; Nl = normal; Abn =  abnormal; CP = chronic pancreatitis; EUS = endoscopic ultrasound; ERCP = endoscopic retrograde cholangiopancreatography; PDAC = pancreatic adenocarcinoma; FU = follow up.

**Figure 2:**
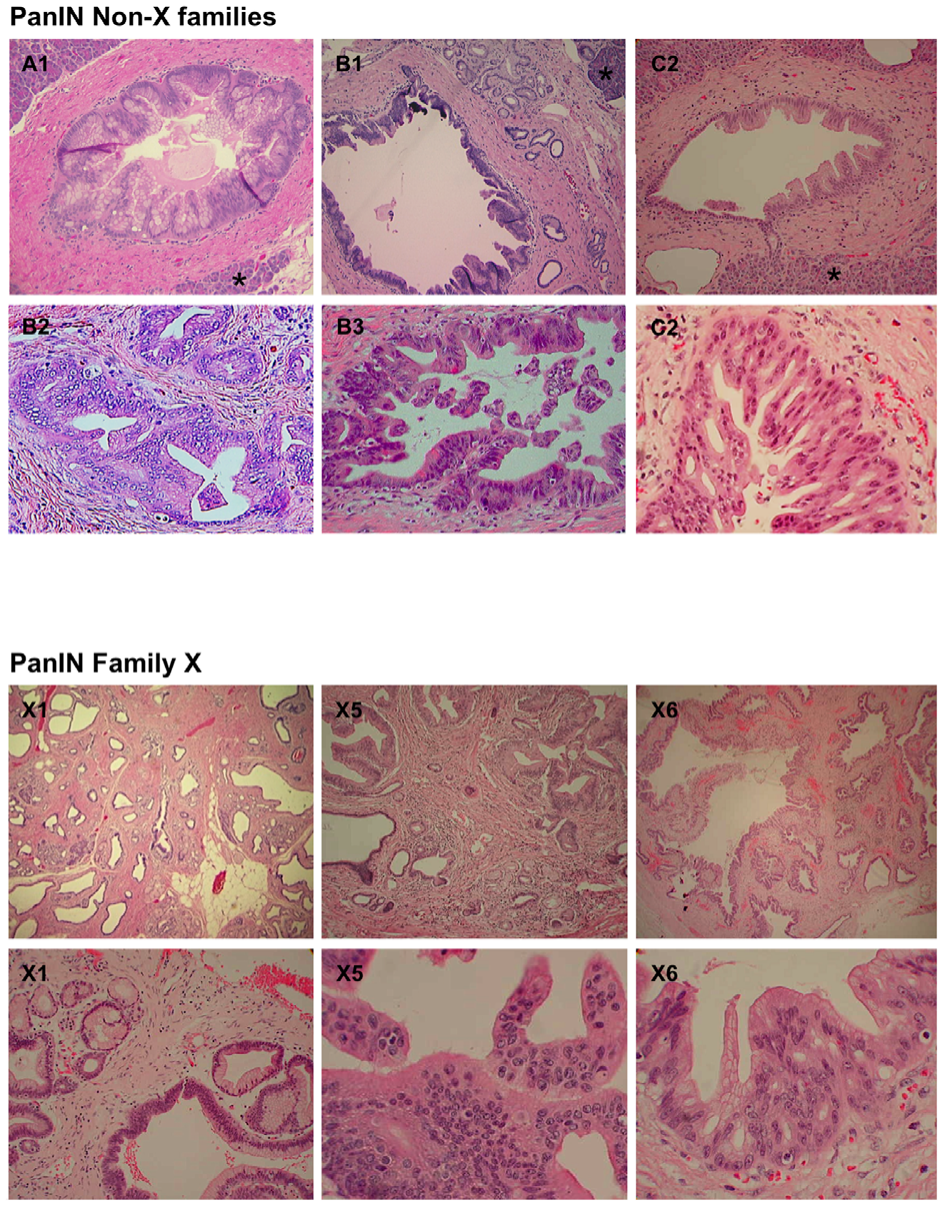
Histology of PanIN lesions. The top panel shows the histology of three members of non-X-families (A1, B1 and C2). Images at the top show PanIN-1 and -2 lesions (magnification ×100); and images at the bottom show PanIN-3 lesions from family B and C; magnification ×200). The lower panel shows the histology of three different members of Family X: X1, X5 and X6 at the top show their gross appearance (magnification ×20); images at the bottom show PanIN-1 from X1 sample (magnification ×100); and PanIN-3 lesions from X5 and X6 (magnification ×200). * indicates adjacent histologically normal appearing tissue.

**Figure 2** shows that familial PanINs resembled sporadic PanINs; however, while areas of adjacent, normal-appearing pancreas (marked by *) were seen in the non-X specimens, pronounced widespread acinar atrophy, fibrosis and multicystic appearance was only seen in Family X.

### Whole transcriptome analysis

Gene expression of 13 PanIN samples was compared to profiling data of whole biopsies from normal donor pancreas (N1 to 4, two replicated samples) and sporadic PDAC (PDAC1 to 6). Unsupervised hierarchical clustering showed a clear separation of samples into four distinct clusters; non-X and Family X PanINs fell into two discrete groups, the former being closer to normal samples, while PDACs formed a single distant cluster (**Figure S1**).

The most commonly up-regulated genes in all PanINs compared to the normal samples were AGR2, S100P, TFF1, LDLR and EMP1, and down-regulated were OLFM4, REG3G, REGL1, and ASNS. When PanIN samples were compared to PDACs, the most commonly up-regulated genes in the cancers were POSTN, COL1A2, SULF1, FN1, IGHM, VCAN and XIST, and these down-regulated were PGC and PPY.

**Figure 3:**
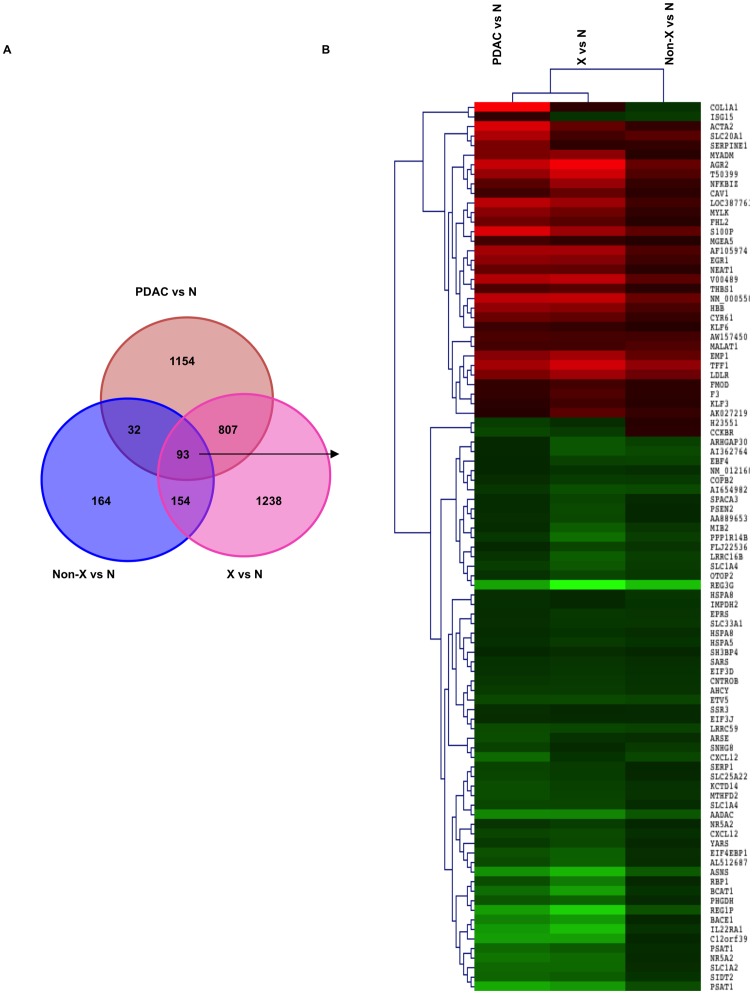
Hierarchical clustering of all the PanIN and PDAC lesions. **(A)** Venn diagram displays the numbers of common and unique probes in PDAC progression; **(B)** Heatmap of 93 commonly dysregulated probes (76 genes) is shown on the right. Each column represents a type of comparison and each row represents a gene probe. The level of up- and down-regulation is represented by the intensity of the red and green colour, respectively.

**Figure 4:**
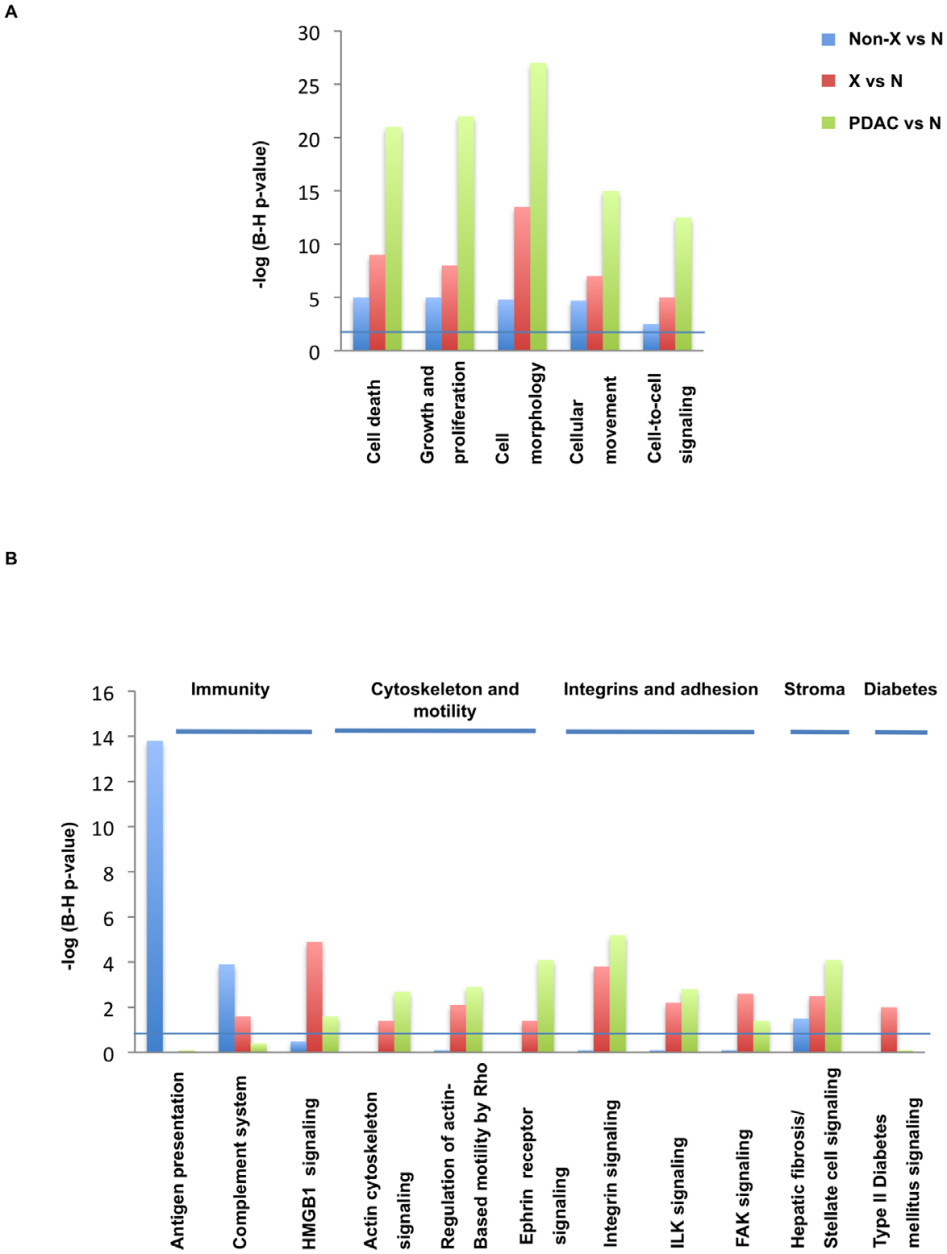
Functional modules and canonical pathways. **(A)** The significant functional modules commonly altered during the transition from normal pancreas to PanIN and PDAC are shown. **(B)** Differences in most significantly affected canonical pathways between PanINs and PDAC samples are presented. The horizontal lines parallel to the x-axis in both images indicate a P = 0.05 threshold.

The Venn diagram in **Figure 3A** shows the total number of gene changes across the three comparisons (non-X, X and PDAC) versus normal pancreas and demonstrates the overall lower number of differentially expressed genes between non-X vs normal pancreas in comparison to Family X vs normal tissue. While this is partially due to the presence of remaining normal-appearing tissues that was more abundant in non-X samples, as the proportion of shared deregulated genes in Family X and sporadic PDACs (900/2292, 39%) was higher than in non-X families (125/443, 28%), this could potentially also underlie the higher aggressiveness of the Family-X PanINs, manifested in their earlier clinical presentation. This was also seen using IPA's Multiple comparison analysis: the most significant ‘Molecular and cellular functions’ which comprise the majority of cancer hallmarks [14] (**Figure 4A**) are all being increasingly affected during neoplastic progression from the PanIN-2/3 to the PDAC and are consistently higher in Family X than in non-X families. Of note, the graphs show the significance of the modules rather than the number of affected genes or direction of their changes.

In addition to shared features, differences between the non-X and X PanINs and PDAC specimens were also seen, particularly in the ‘seventh hallmark of cancer’, immune functions [15], [16]. The most affected canonical pathways are shown in **Figure 4B**. The highest ranked ‘Antigen Presentation’ was predominantly affected in non-X families, with the decrease in the key components of antigen-presenting machinery (CD74, HLA-A/B, HLA-DMA, HLA-DRA/B1, HLA-DQA1/B1, HLA-DPA1/B1). In contrast, antigen presentation in Family X appeared more similar to the normal pancreas, while PDAC was characterised by up-regulation of HLA-B, HLA-DPA1, HLA-DQA1, HLA-DRB4, TAP2, as well as genes involved in migration of antigen presenting cells i.e. DMBT1 and CD29, which are not changed in familial PanINs. Dendritic cell migration and macrophage recruitment were also down-regulated in non-X families (decrease in CXCL12, CXCR4, VCAM-1 and ICAM2 expression).

**Figure 5:**
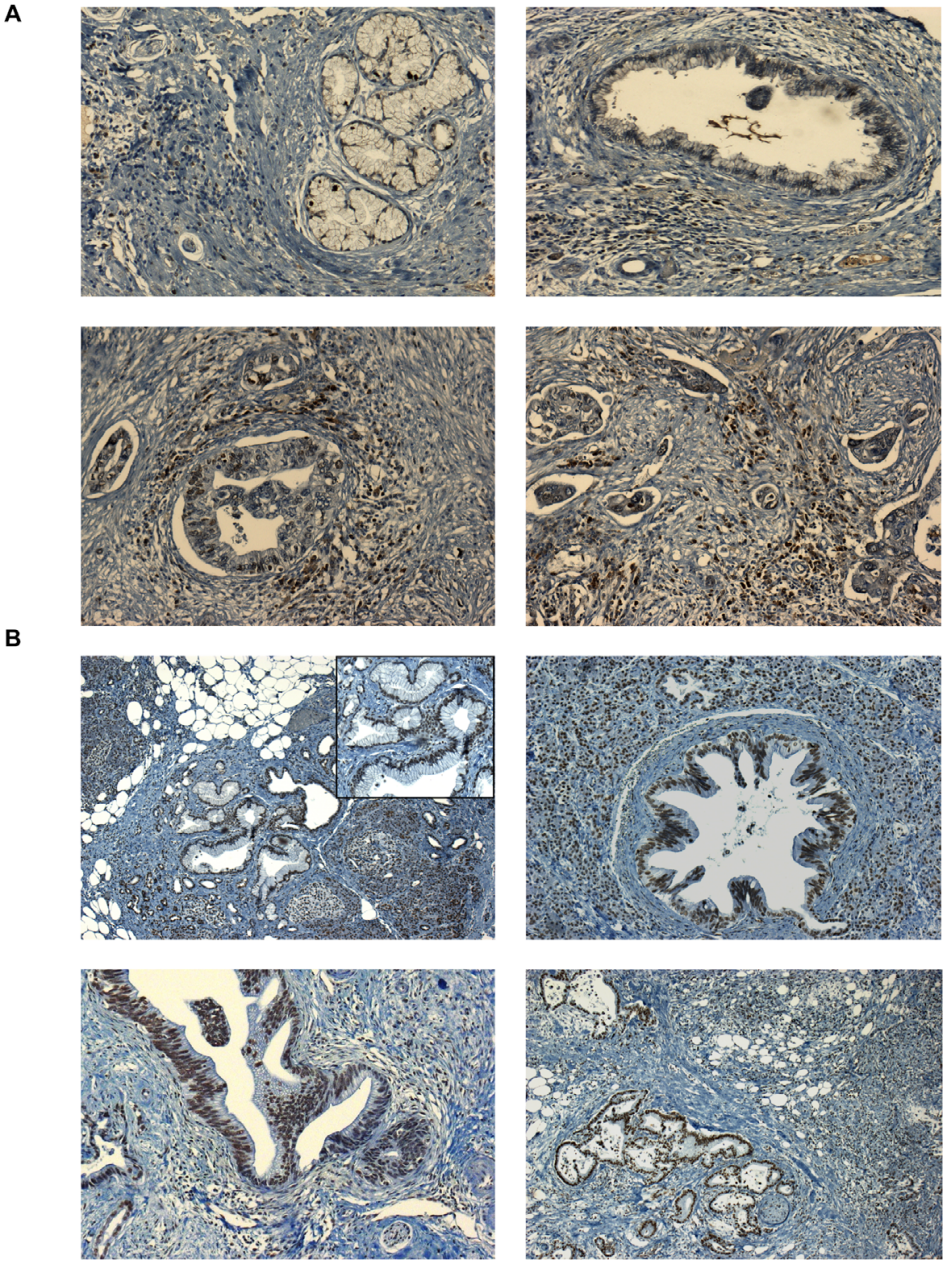
Localisation and expression of BCL6 and HMGB1. **(A)** Representative images of BCL6 positive cells (brown staining) in the stroma in the vicinity of PanIN lesions are shown in the top two panels (both magnified ×100); two bottom images show inflammatory infiltrate with BCL6 immunoreactive cells in two PDAC cases (magnification ×100 and ×50, respectively). **(B)** HMGB1 nuclear expression (brown staining) was seen in all pancreatic compartments, including stromal immune infiltrate: top panels show PanIN-1 (left) and -2 (right) (magnification ×50, insert and second panel x100); bottom panels show PanIN-3 (left) and PDAC (right) (magnification ×100 and ×50, respectively).

Humoral immune response was also significantly affected in non-X families, with lower expression of RGS1 (B cell development, activation and proliferation [17]) and complement system, namely C3, C1QA/B/C, C4B, CFB and SERPING1 (**Figure 4B**). In contrast, EBF1, IL7R and PRDM1/BLIMP1 were up-regulated only in Family X, while up-regulation of POU2AF1 and BCL6 (B cell growth, maturation and formation of germinal centres) [18]) was seen in both PanIN-X and PDAC samples. In PDAC only, C3, C1S, and CD55 were up-regulated, and PDAC was characterised by a strong pro-inflammatory response (up-regulation of TGFB1, TGFBR1, STAT2, STAT6, SPP1, LIF). As BCL6 was recently shown to be one of the 12 stromal genes that can distinguish preinvasive from invasive disease in esophageal carcinoma based on profiling of the microdissected stroma only [19], we have selected this gene for validation in pancreatic tissues. We show that the increased level of BCL6 transcript (>2 fold) seen in PanIN-X and PDAC, is contributed by strong nuclear immunoreactivity in the stromal inflammatory cells (**Figure 5A**). Of 24 PanIN lesions on TMA1, 16 (67%) displayed inflammatory infiltrate, nine of which (56%) (2/2 PanIN-1, 4/7 PanIN-2 and 3/7 PanIN-3) comprised BCL6 positive cells. Of 15 PDAC cases, 13 (87%) comprised inflammatory infiltrate; 11 of these (85%), including four cancers with PanINs, showed BCL6 immunoreactivity.

A prototypic damage-associated molecular pattern (DAMP) of HMGB1/2 signalling was also evident, predominantly in PanIN-X and PDAC (**Figure 4B**); this pathway signals through RAGE [20]. To establish the localisation and expression of HMGB1, IHC analysis was performed (**Figure 5B**). The nuclear immunoreactivity was seen in both the exocrine and endocrine cells, as well as in the immune stromal component. HMGB1 expression was seen in 67% (16/24) of PanINs (3/4 PanIN-1, 5/10 PanIN-2 and 8/10 PanIN-3) from TMA1, in all PanINs found within 15 PDAC cases and in 13/15 (87%) PDAC lesions (two PDAC cases with no/weak expression were poorly differentiated). Of note, HMGB1 expression has already been associated with various cancer diseases (for review see [21]). In PDAC, serum HMGB1 was recently reported to correlate with stage, resectability and early vs late PDAC [22].

The DAMP superfamily includes also S100 genes, several of which were up-regulated in PanIN-X: S100A4, S100A6, S100A7A, S100A10, S100A13 and S100A16. Deregulation of S100A2 and S100A11 was additionally seen in PDAC, while S100P was commonly up-regulated in all PanIN lesions (X and non-X) and PDACs, further substantiating the importance of S100 genes in the development and progression of FPC as seen in sporadic cases [23], [24]. Further canonical pathways whose significance increased with cancer progression were cytoskeleton and motility/invasion (‘Actin cytoskeleton signaling’, ‘Regulation of actin-based motility by Rho’ and ‘Ephrin signaling’) (**Table S1**); adhesion (‘Integrin signaling’, ‘ILK signaling’ and ‘FAK signaling’) (**Table S2**); and stromal response, with higher expression and number of ECM genes seen as PanINs progress to PDAC (**Figure 4B**). A large number of these clustered within ‘Hepatic fibrosis and stellate cell activation’ (**Table S3**) and can potentially represent activation of pancreatic stellate cells, which have been reported to have highly similar profiles to hepatic stellate cells [25]. Furthermore, in addition to COL1A1, COL1A2 and COL3A1, other collagens (COL4-6A2 and COL12A1), and ECM genes (BGN, VCAN, DCN, SPARC, SPON1 and THBS1) were up-regulated in PanIN-X lesions and PDACs; in PDACs, stromal involvement was characterised by an even higher diversity of collagens (COL10A1, COL11A1, COL14-16A1 and COL18A1 were additionally seen), and even higher expression than in PanIN-X lesions of the ECM genes mentioned above. Importantly, COL11A1 was recently shown to be a specific marker of pancreatic stellate cells [26], indicating that their accumulation is particularly pronounced in PDAC. Significant accumulation of matrix metallo-proteinase and cysteine proteinases was seen already in PanIN-X lesions,, with increased levels of MMP1, MMP2 and MMP19, and CTSC, CTSE and CTSK, respectively. Additional proteases (MMP7, MMP9, MMP11, MMP14, MMP28 and CTSA and CTSB) were deregulated only in PDAC specimens.

Family X samples differed from both non-X PanINs and PDACs in the deregulation of a number of genes involved in insulin signalling and diabetes (INSR, IRS1 and 2, IGF1, IGFBP2-BP7, CASR, ADIPOQ and NR5A2); this is illustrated in **Figure S2 and S3**, with accompanying discussion).

### Comparison of familial PanINs to data from sporadic PDACs

**Figure 6:**
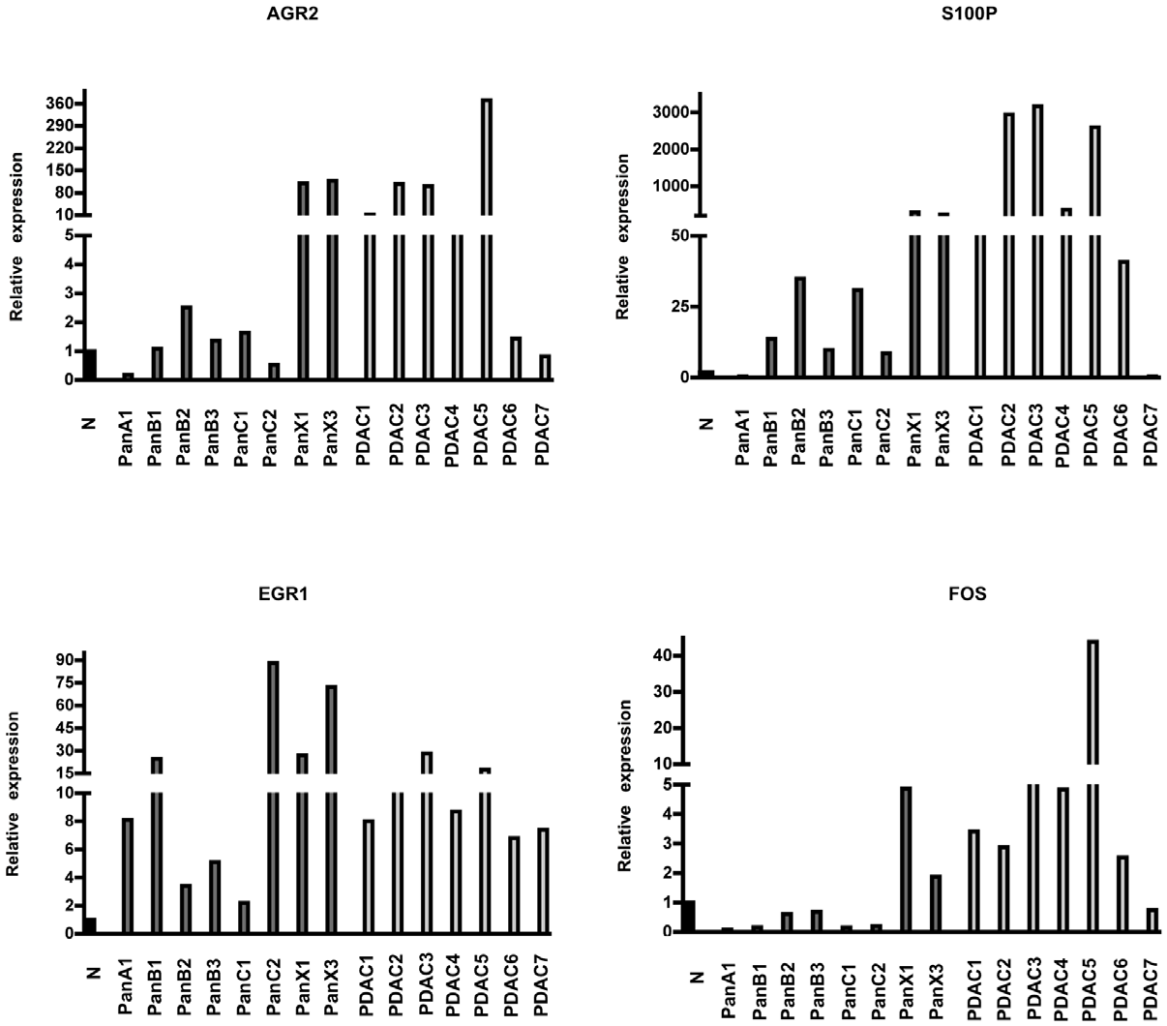
Confirmation of gene expression profiling. QRT-PCR analysis validated the differential expression for AGR2, S100P, FOS and EGR1 in primary PanIN lesions: PanA1, B1-3 and C1,2 represent non-X families, while PanX1 and 3 belong to Family X samples; PDAC1-7 represent seven different PDAC specimens.

Multiple comparisons of our data with previously published reports were achieved using Pancreatic Expression database (http://www.pancreasexpression.org/). When data from non-X and X PanINs were compared to published profiles from microdissected PanINs from sporadic PDACs [5], [27], 44 and 185 deregulated genes were shared, respectively (**Table S4**). Three of the commonly deregulated genes were successfully validated by QRT-PCR: AGR2, S100P and EGR1. In addition, higher expression of FOS in Family X PanINs and majority of PDACs was also confirmed (**Figure 6**).

When non-X and X PanIN data were compared to dissected and bulk sporadic PDAC profiles (references are listed in Pancreatic Expression database), around 5% and 25% of overlap was seen, respectively. This is less than was seen in our PanIN vs PDAC comparison, and it is largely attributed to different platforms and experimental techniques used.

Lastly, non-X and X PanIN transcriptomes were compared to our PanIN-3 proteome [12], 46/900 (5%) and 132/900 (15%) proteins matched the RNA transcripts, respectively; these data independently validate our findings at the protein level (**Table S5A** and **B**).

### Common PanIN-PDAC progression gene signature

The heatmap in **Figure 3B** and **Table S6** display the 93 probes representing 76 commonly differentially expressed transcripts across all comparisons (X vs Normal, non-X vs Normal, PDAC vs Normal).

More than 10 different enzymes were commonly down-regulated, including the ones involved in synthesis and catabolism of amino acids (ASNS, BCAT1, EPRS, SARS, YARS, PSAT1); moreover, deregulation of several solute carriers points to an impaired amino acid transport (SLC1A2, SLC1A4, SLC25A22). Expression changes in SLC20A1, a sodium-dependent phosphate symporter, and KCTD14 (potassium channel tetramerisation domain-containing-14) suggests impaired ion transport as well.

Commonly expressed stromal genes include COL1A1, THBS1, FMOD and SERPINE1 across all PanIN lesions and PDAC. COL1A1, a major component of pancreatic desmoplasia promotes invasion and metastasis in PDAC [28]. Increased expression of THBS1 is a prognostic predictor of increased invasiveness in PDAC [29] and correlates with the progression of metaplasia-dysplasia and cancer in oesophagus [19]. In addition, THBS1 also increases the expression of SERPINE1 [30], which is modestly up-regulated in both non-X and X PanINs and strongly up-regulated in PDAC. Therefore, while these stromal genes have previously been associated with PDAC, we show that they are already over-expressed in PanIN lesions prior to cancer formation.

One of the key regulators of the NFkB pathway, NFKBIZ, is seen deregulated in all PanINs. Its up-regulation may contribute to increased inflammation in the pancreas that favours tumour progression [31]. Expression of an interleukin receptor, IL22RA1, was decreased in PanINs and PDACs, but as this gene is mainly expressed in the islet cells [32], islet cell loss could potentially explain such result. CXCL12 was also commonly affected; it plays a role in cancer spread/metastases via interaction with its receptor CXCR4. Both CXCL12 and CXCR4 were down-regulated in non-X PanINs, while only CXCL12 is down-regulated in the PanIN X lesions, and the CXCR4 levels are normal. In contrast, PDACs showed increased levels of CXCR4; this is a well-established feature of many cancer types [33] and is a predictor of poor survival in PDAC [34].

Several transcriptional factors were also found up-regulated in both PanINs and PDACs: KLF3, KLF6 and EGR1.

Finally, REG3G and REGL, markers for pancreatic injury [35] were consistently under-expressed in both PanINs and PDACs, and indicate loss of acinar cells during PDAC development.

**Figure 7:**
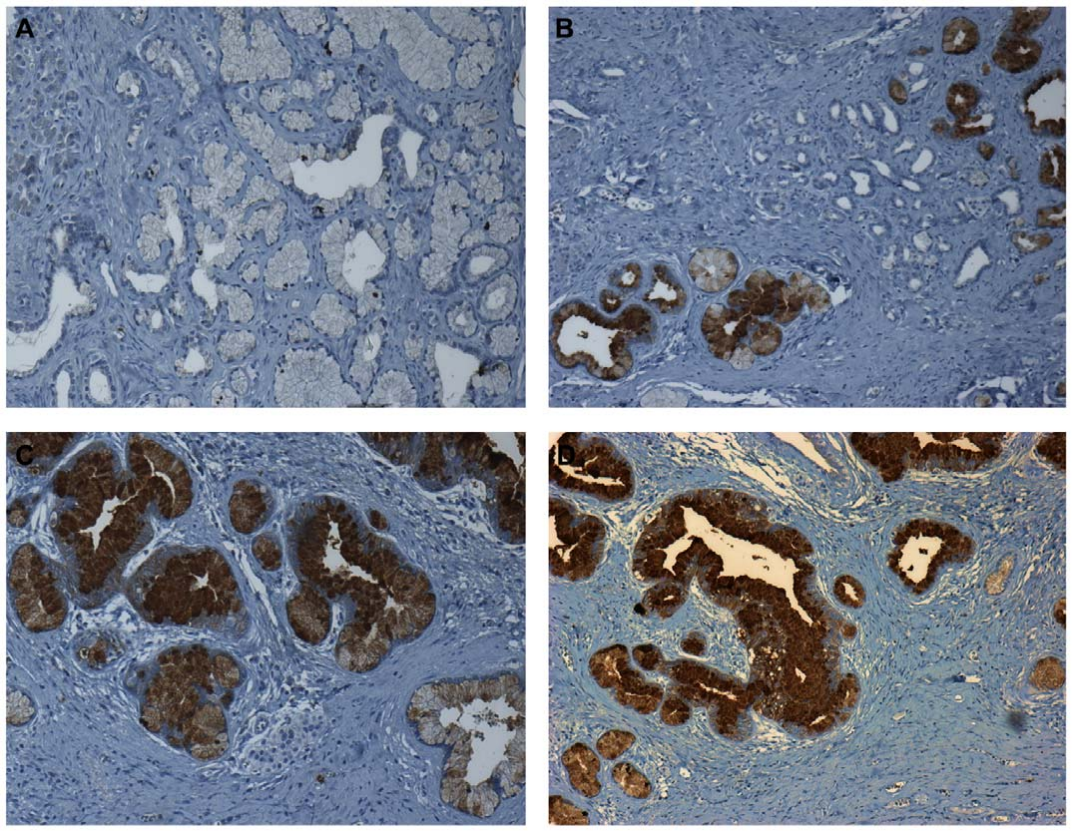
Expression of TFF1 in familial PanIN lesions. Panel **(A)** shows PanIN-1 with no TFF1 immunoreactivity, **(B)** and **(C)** PanIN-2 and **(D)** PanIN-3 lesion in the centre with strong TFF1 expression (all magnified x100).

*In silico* comparison of our 76 commonly differentially expressed PanIN/PDAC transcripts with our sporadic and familial PanIN-3 proteomics data ([12], and unpublished data) highlighted the expression of nine of the 76 genes also at the protein level: ACTA2, AGR2, AHCY, COL1A1, COPB2, HSPA5, HSPA8, S100P and TFF1, providing thus an independent validation of our profiling data. While we have previously shown that AGR2 is expressed in both sporadic and familial PanINs [36], we here additionally validated TFF1 by IHC using sections derived from Family X tissues (**Figure 7**). This demonstrated the almost universal expression of TFF1 in the familial precursor lesions (5/10 PanIN-1, 9/9 PanIN-2 and 4/4 PanIN-3) as shown previously in sporadic cases [37].

## Discussion

While early description of PDAC precursor lesions dates back to the 1950's [38], and the hypothesis that atypical hyperplasia and carcinoma *in situ* are precursors for PDACs is more than 30 years old [39], the consensus PanIN nomenclature was established relatively recently [3].

Despite several detailed histological and clinical studies [40], and a report of increased prevalence of PanIN lesions in both sporadic [41] and familial PDAC patients [42], our knowledge of the underlying molecular events in these precursor lesions is still limited. This is largely due to the inaccessibility of PanIN specimens.

Here, we have analysed PanIN-2/3 lesions from pancreatectomy specimens derived from FPC *without* the presence of invasive carcinoma, which is critical as it is often difficult to distinguish between true PanIN-3 lesions and cancerisation of ducts by well-differentiated invasive cancer in the specimen that contains both. Only two similar small-scale studies have been reported on PanIN lesions detected in the absence of cancer: Zhang et al [43] analysed KRAS mutations and protein expression of p53, p16 and cyclin D1 in PanINs in tumour-free heterotopic pancreas of PDAC patients and Baumgart et al [44] analysed PanIN samples from a patient with chronic pancreatitis that had PanIN-3 lesions; both studies provide direct evidence for the PanIN-PDAC progression model. The comprehensive molecular analyses presented here not only provides definitive support of the progression model, but also enabled us to investigate the underlying molecular pathways and to assess the similarities between the sporadic and familial precursor lesions on a genome-wide scale.

While the similar prevalence of ‘signature’ mutations in sporadic and familial PDAC specimens has previously been reported [45], and mutation analyses in Family X conformed to these data [6], we now show that on the transcriptome level, PanIN lesions in our familial cases have also undergone similar changes as those seen in sporadic cancer. Based on comparison of data from SEER (Surveillance Epidemiology and End Results) database and data on familial PanINs from Brune et al [46] Schwartz and Henson [47] suggested that familial PDACs may have similar or overlapping pathways to those of sporadic cases. We now provide molecular evidence that this is indeed the case.

Two transcriptome studies of sporadic PanINs in the setting of pancreatic cancer have been reported; Prasad et al [27] compared microdissected PanIN-1B/2 lesions with normal ducts; Buchholz et al [5] used microdissected material to compare PanINs of all grades to normal ducts and PDACs, showing a steady increase in number of differential transcripts with advanced dysplasia. These studies used custom-made cDNA and oligo-based arrays, respectively, with amplified fluorolabelled material from sporadic PDACs; we used much larger coverage Affymetrix arrays with the unamplified material from enriched primary PanIN-2/3 lesions that occurred in the absence of cancer. The overlap between the genes across the three studies was around 4% for non-X PanINs and 18% for Family X PanINs. Considering the limited congruence reported between profiling studies [48] and the differences between the three data sets, this is in fact a fair amount of overlap. The discovery of common genes through such disparate studies proves that these are robust genes uniformly deregulated during PanIN progression.

Based on the volume of transcriptional changes in PanINs derived from Family X and non-X families, with around 40% of shared differential transcripts with PDAC, the PanIN lesions in Family X appeared more molecularly attuned to cancer. These transcriptional changes mirror both the histopathological and the clinically more aggressive picture, as PDAC in Family X develops ∼20 years earlier (median 40 years of age) and cancer in non-X families 5–10 years earlier (median 54 years) than in sporadic cancer.

The most pronounced differences between the PanIN lesions from Family X and non-X families were seen in the immune response: while the inflammatory response in non-X families was generally deficient, with significant underrepresentation of genes in the antigen presentation and humoral response pathways, the immune response profile in Family X was in many aspects similar to PDAC. Interestingly, up-regulation of KIT, its ligand KITLG/SCF and tryptase TPSAB1 were seen already in Family X PanINs. KIT is a proto-oncogene associated with several tumours that enhances proliferation and invasion of pancreatic cancer cell lines [49]; KITLG/SCF and TPSAB1 are mast cell markers indicating an early infiltration of stroma surrounding PanINs with mast cells. A pro-inflammatory milieu composed of macrophages and mast cells has been shown to promote cancer growth and invasion [50], [51]; mast cell inhibition has been proposed as a therapeutic strategy for PDAC [52].

A further noticeable difference seen between the PanINs reflects the Family X histology, which shows an extensive desmoplastic change; this was characterised with both higher expression and increased numbers of stroma-associated genes, indicating that the stroma co-evolves with epithelial elements in the PDAC precursor lesions. Similar findings have been reported in breast precursor lesions, where extensive gene expression changes in the stroma are associated with ductal carcinoma in situ (DCIS) [53]. Further significant increases in expression of stromal genes were seen in our PDAC data; the importance of the stroma in biology of sporadic pancreatic cancer is well recognised [54], [55]. All together, these data indicate that co-operating transcriptional changes in both tumour and its microenvironment can dramatically alter the natural history of the disease and that monitoring both compartments might also provide a better predictor of pancreatic cancer evolution in the familial setting.

Seventy-six common genes were uniformly affected throughout the disease evolution and appear to be fundamental to neoplastic progression in pancreatic cancer regardless of whether it is sporadic or familial. Within this gene set, we highlight two genes, S100P and AGR2, which with COL1A1 show the highest over-expression in both PanIN and PDAC lesions in the current analysis as well as in our previously published data. Both have already been involved in development and progression of sporadic PDAC: S100P expression increases with the PanIN grade [24], and AGR2 is uniformly expressed from earliest PanIN-1 lesions [36], [56]. As these two proteins are expressed also in familial lesions they represent promising diagnostic, preventive and therapeutic targets.

The 76 common genes observed are markers of dysplastic lesions that underlie neoplastic progression, we thus propose that this core neoplastic profile might be useful in monitoring of PanIN progression and could form a basis for the design of a molecular test to be used in conjunction with EUS/MRCP surveillance. While the number of affected members in the kindred increases the risk of PDAC development, given the varying risks for PDAC between kindreds with heterogenous clinical syndromes, it is challenging to differentiate the patients who will develop rapid neoplastic progression from the ones with the stable disease and to identify which groups will benefit most from a comprehensive pancreatic cancer surveillance program. Currently, if abnormal EUS and MRCP findings warrant a tissue diagnosis, a partial pancreatectomy with detailed histopathological examination is performed. Inclusion of an additional, sensitive molecular assay, could be instrumental in the decision on how to proceed (further surveillance versus pancreatectomy). Moreover, this transcriptome profile could be used in conjunction with needle biopsies of indeterminate lesions of the pancreas in high-risk settings to identify progressing PanIN-3 lesions.

Nine of the obtained 76 PanIN/PDAC common genes: were independently validated by *in silico* comparison with our PanIN-3 proteomics data: ACTA2, AGR2, AHCY, COL1A1, COPB2, HSPA5, HSPA8, S100P and TFF1. Protein validation of abnormally expressed transcripts was also performed by IHC (AGR2, TFF1) and CYR61, detected in our transcriptome studies, was recently reported to be expressed in (sporadic) PanIN and PDAC samples [57]. It will now be critical to further confirm the validity of our proposed panel in an independent set of familial/sporadic cases ideally through multi-center retrospective studies. In that respect, it is of great importance that another three genes seen in our transcriptome PanIN data are shown to be a part of a six gene signature recently reported to predict survival of PDAC patients [58]: FOSB was over-expressed in Family X PanINs and was just below the significance cut off in non-X families, and NFKBIZ and KLF6 are within our common 76 gene set.

The major limitation of the present study is a relatively small number of samples analysed as well as the paucity of the obtained material; the infrequent availability of primary PanIN lesions in the absence of pancreatic cancer, limits a more extensive validation of the results. Also, for gene expression profiling, we used enriched (macrodissected), rather than microdissected samples in order to both avoid amplification of the material and to be able to assess the stromal and immune response to PanIN growth (as these are increasingly highlighted as critical to cancer progression [19]), although we were aware that this would preclude the assignment of expression changes to individual stage of PanINs. Both of these limitations were, however, circumvented by extensive analyses and comparisons to both dissected and bulk pancreatic specimens using the most recently updated version of Pancreas Expression database [59] which allowed us to integrate our results and present them in the context of previously reported data, as well as to perform large-scale *in silico* validation of our results. Of note, all the data sets obtained in our study will (upon publication) be included into our database and made available for further mining to wider pancreas cancer research community.

In summary, our comprehensive analysis of unique clinical specimens provides sufficient evidence to support the concept that the PanIN-2/3 lesions are true non-invasive precursors of PDACs, that familial precursor lesions share the fundamental signalling pathways seen in sporadic pancreatic cancers, and that it is the accumulation and the volume of concomitant changes at the transcriptome level in both in the epithelial and stromal compartments, as well as the pro-inflammatory milieu that dictate the speed of progression of PanINs to PDAC also in the familial setting.

## Materials and Methods

### Ethics Statement

All specimens used in this study were collected with patient consent and under protocols approved by the Institutional Review Board at the University of Washington (Seattle, Washington, United States).

### Sample preparation and RNA isolation

13 PanIN specimens from pancreatectomies were analysed: six originated from non-X (one from Family A, three from Family B and two from Family C) kindreds and seven were from four different patients from Family X. Further four specimens from two donor pancreata and six sporadic PDACs were also used. Of note, sporadic PDAC samples were used due to unavailability of the matched cancer samples from the analysed families, as profiled lesions were obtained before cancer has developed (in cases where members of the family did present with cancer, it was already in an advanced stage, so patients were not amenable to surgery and the samples could not be procured).

In order to preserve the stromal response which is critical for growth of PDAC, we have not microdissected the specimens; however, following detailed histopathological analyisis, frozen tissue blocks that comprised most pronounced multifocal PanIN-2 with focal PanIN-3 lesions were selected for RNA isolation. While PanIN lesions from non-X family samples were often surrounded by histologically normal-appearing acinar cells with PanIN lesion cellularity around 30–40%, Family X samples were characterised by much more widespread precursor lesions, with cellularity around 60%. The estimated percentage of high grade PanINs in the specimens was around 10–15%. PDAC specimens were classified as T2N1M0 and comprised >60% of cancer cells.

Total RNA was isolated using TRIzol reagent (Invitrogen Life Technologies) according to the manufacturer's protocol. The quantity and quality of the samples were assessed using Thermo Scientific NanoDrop 1000 spectrophotometer and Agilant 2100 Bioanalyzer, respectively.

### Expression profiling and data analysis

Expression profiling was performed using GeneChip HG-U133 set of arrays (Affymetrix, Santa Clara, CA, USA). Ten micrograms of total RNA was reverse transcribed, biotin-labelled, fragmented and hybridized to arrays, all according to the manufacturer's instructions (Affymetrix, Santa Clara, CA, USA). The arrays were scanned with a GeneChip scanner 3000 7G. After scanning, raw. CEL files were analyzed using Bioconductor (http://www.bioconductor.org/) packages within the open source R statistical environment (www.r-project.org). After quality control inspection, data were normalised jointly using the Robust Multi-array Average (RMA) algorithm and filtered using standard deviation calls to select the most deregulated probes across all experiments. To account for technical replicates, the “duplicateCorrelation” function was used [60]. Genes differentially regulated between the biological groups were identified using limma [61]. The Benjamini and Hochberg (BH) false discovery rate was used for multiple testing corrections; a double cut-off of false discovery rate (FDR) <0.05 and unlogged fold change of ≥2 was used.

### Quantitative Real-Time PCR (QRT-PCR)

Deregulation of AGR2, S100P, EGR1 and FOS was validated using TaqMan probes AGR2/Hs00180702_m1; S100P/Hs00195584_m1; EGR1/ Hs00152928_m1 and FOS/Hs00170630_m1 from Applied Biosystems (Foster City, CA, USA). One µg of total RNA was reverse transcribed and triplicated PCR reactions carried out on ABI 7500 system. 18S was used as endogenous control. Data were analyzed using SDS version 1.3 (Applied Biosystems).

### Pathway analysis

Ingenuity Pathway analysis (IPA) is a web-based application (www.Ingenuity.com) that enables building of functional modules and biological networks based on entered data and published associations that are compiled, manually curated, and stored in the IPA knowledge database, IPKB. Differentially expressed genes were interrogated and scores for each generated network reported, thus ensuring that contributing genes are not selected by random chance (for example, a score of 2 gives 99% confidence, with higher scores signifying greater confidence). Thus, IPA prioritizes the networks, identifies the associated genes, and assigns the most significant biological functions and corresponding canonical pathways to each network. The global functional analysis calculates this significance using the right-tailed Fisher's exact test, with a *P* value <0.05 being significant.

### Immunohistochemistry

Validation of BCL6 and HMGB1 was performed on tissue microarray (TMA1) comprising 32 cores representing 21 different cases comprising 24 sporadic PanIN lesions (four PanIN-1, ten PanIN-2 and ten PanIN-3), as well as on 15 sporadic PDAC cases (within these, seven PanIN-1, four PanIN-2 and five PanIN-3 were found). In addition, validation of TFF1 was performed on TMA2 comprising 60 cores representing 11 different FFPE tissue blocks from Family X members (ten PanIN-1, nine PanIN-2 and four PanIN-3). Immunohistochemical staining was performed on 4 µm paraffin-embedded tissue sections using the Ventana DiscoveryTM System, Illkirch, France (www.ventanadiscovery.com) following the manufacturer's protocols. Following antibodies were used: rabbit polyclonal anti-BCL6 antibody (HPA004899) from Sigma-Aldrich (1:30 dilution), rabbit polyclonal anti-HMGB1 antibody (ab18256) from Abcam (1:1000 dilution), and rabbit polyclonal anti-estrogen inducible protein pS2 (TFF1) antibody (ab50806) from Abcam (1:100).

## Supporting Information

Figure S1 **The dendrogram shows relationships within 20 pancreas tissue samples (two normal donor pancreata with one replicated sample, 13 PanINs and four PDACs) based on top 12,000 most variable genes.** Normal pancreas is denoted N, PanINs in Family X and PanINs in non-X families are denoted as PanX and PanA-C, respectively, and pancreatic cancer is denoted as PDAC. Of note, two PDAC and a replicate of one normal specimen were removed during the hybridisation quality assessment.
(TIF)

Figure S2 **Affected genes within Diabetes mellitus signaling pathway.**
(TIF)

Figure S3 **Accompanying data for Figure S2.**
(DOC)

Table S1 **Deregulated genes involved in actin cytoskeleton and motility.**
(XLS)

Table S2 **Deregulated genes involved in integrin signaling and adhesion.**
(XLS)

Table S3 **Fibrosis and stellate cell response.**
(XLS)

Table S4 **Comparison with published sporadic PanIN gene expression profiles.**
(XLS)

Table S5 **Comparison of non-X (A) and PanIN-X samples (B) to PanIN proteomics data.**
(XLS)

Table S6 **93 probes representing 76 commonly expressed genes in all PanIN and PDAC samples.**
(XLS)
