# Supplementary figures and images for: Molecular Analysis of Precursor Lesions in Familial Pancreatic Cancer
Source: PLoS One. 2013 Jan 23;8(1):e54830. doi: 10.1371/journal.pone.0054830 (PMC3553106; doi:10.1371/journal.pone.0054830)

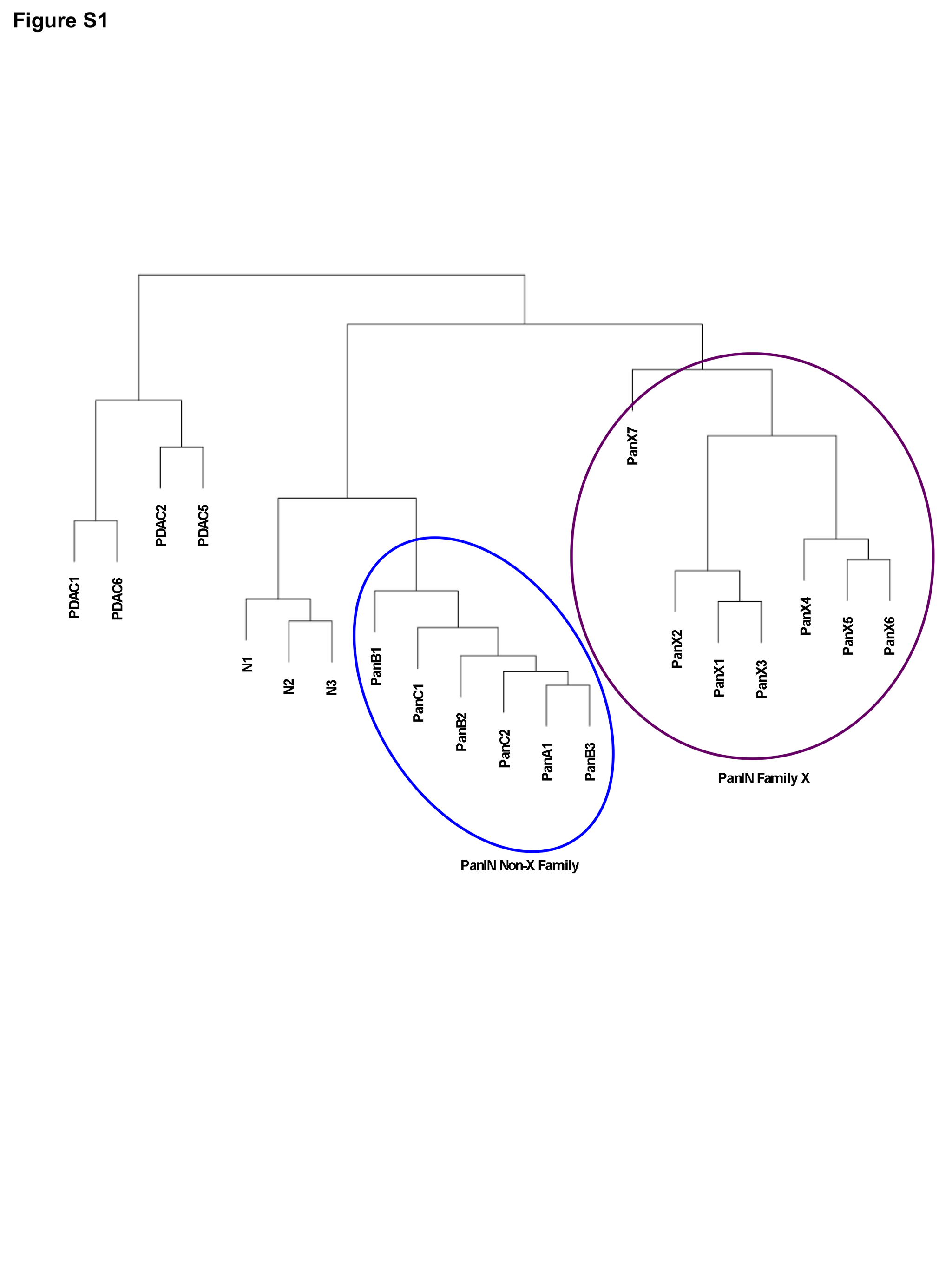

Supplement: Figure S1 — The dendrogram shows relationships within 20 pancreas tissue samples (two normal donor pancreata with one replicated sample, 13 PanINs and four PDACs) based on top 12,000 most variable genes. Normal pancreas is denoted N, PanINs in Family X and PanINs in non-X families are denoted as PanX and PanA-C, respectively, and pancreatic cancer is denoted as PDAC. Of note, two PDAC and a replicate of one normal specimen were removed during the hybridisation quality assessment. (TIF) [file pone.0054830.s001.tif]

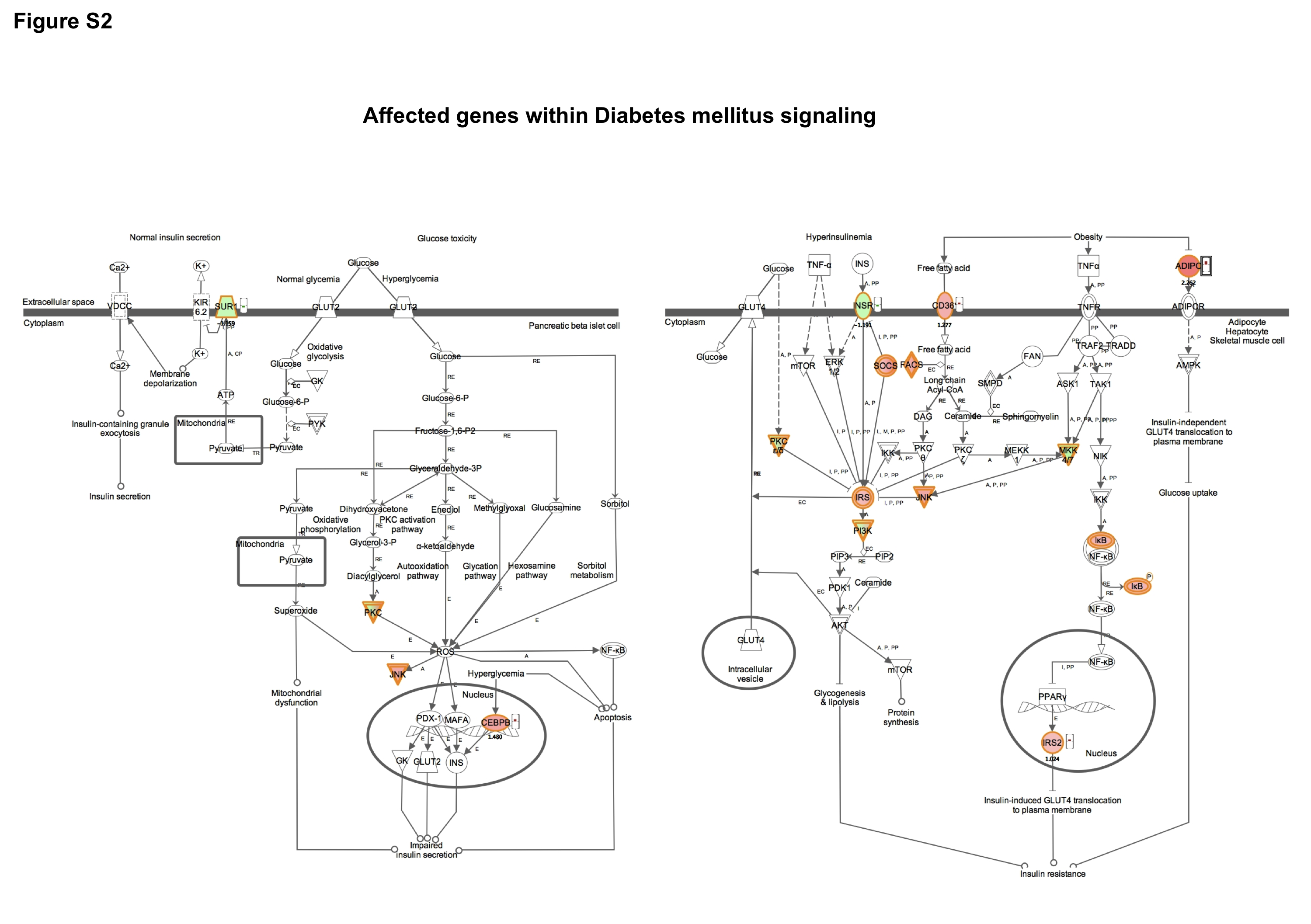

Supplement: Figure S2 — Affected genes within Diabetes mellitus signaling pathway. (TIF) [file pone.0054830.s002.tif]
