# Supplementary material for: Molecular Analysis of Precursor Lesions in Familial Pancreatic Cancer
Source: PLoS One. 2013 Jan 23;8(1):e54830. doi: 10.1371/journal.pone.0054830 (PMC3553106; doi:10.1371/journal.pone.0054830)
Supplement: Figure S3 — Accompanying data for Figure S2. (DOC) [file pone.0054830.s003.doc]

**Figure S2** accompanying data:

The PanIN X profile was characterised by a large number of genes implicated in Diabetes mellitus (DM), some of which are illustrated on **Figure S2** (red colour indicates up-regulation, and green down-regulation). These could underlie a strong association of this kindred with DM. Namely, three subjects from this family (II.1, III.4 and III.17) had insulin-dependent diabetes mellitus (IDDM) for over 10 years before they were diagnosed with PDAC (it has been reported that diabetic patients diagnosed at least 10 years prior to PDAC have a statistically significant 50% increased risk of developing it compared to non-diabetic controls) (1). In four family members IDDM was diagnosed with the malignancy (I.1, II.5, II.6 and III.15); III.2 developed DM at the age of 32, and one subject died in diabetic ketotic coma at 27 years of age (III.3).

The association between diabetes and PDAC is well established (around 60% of patients with histologically proven pre-cancer or cancer have an abnormal glucose tolerance test or diabetes), although it is still uncertain whether DM is a true etiologic factor or a consequence of PDAC during a pre-diagnostic stage [1]. Experimental data showing that PDAC resection improves glucose tolerance and leads to resolution of DM in 57% of patients after pancreaticoduodenectomy [2], and that supernatants of PDAC cell lines induce glucose intolerance in SCID mice suggest that DM in PDAC could be a paraneoplastic phenomenon [3]. We now provide a set of potential candidate genes with diabetogenic characteristics in PanIN lesions from Family X that could be explored further in the context of diabetes in the sporadic form of the disease. From a clinical standpoint, it might be valuable to develop biomarker(s) that could distinguish between DM in the setting of PDAC from ‘classical’ DM, which might then be used for screening the adult population with new onset DM [4].

1. Silverman DT, Schiffman M, Everhart J, Goldstein A, Lillemoe KD, Swanson GM et al. (1990) Diabetes mellitus, other medical conditions and familial history of cancer as risk factors for pancreatic cancer. Br J Cancer 80:1830-1837.

2. Pannala R, Leirness JB, Bamlet WR, Basu A, Petersen GM, Chari ST. (2008) Prevalence and clinical profile of pancreatic cancer-associated diabetes mellitus. Gastroenterology 134:981-987.

3. Chari ST, Leibson CL, Rabe KG, Timmons LJ, Ransom J, de Andrade M et al. (2008) Pancreatic cancer-associated diabetes mellitus: prevalence and temporal association with diagnosis of cancer. Gastroenterology 134:95-101.

4. Pannala R, Basu A, Petersen GM, Chari ST. (2009) New-onset diabetes: a potential clue to the early diagnosis of pancreatic cancer. Lancet Oncol 10: 88-95.
